# Surfactin Mitigates a High-Fat Diet and Streptozotocin-Induced Type 2 Diabetes through Improving Pancreatic Dysfunction and Inhibiting Inflammatory Response

**DOI:** 10.3390/ijms231911086

**Published:** 2022-09-21

**Authors:** Xiaoyu Chen, Hongyuan Zhao, Yajun Lu, Huawei Liu, Fanqiang Meng, Zhaoxin Lu, Yingjian Lu

**Affiliations:** 1 College of Food Science and Technology, Nanjing Agricultural University, Nanjing 210095, China; 2 College of Chemistry and Chemical, Nanjing Tech University, Nanjing 211816, China; 3 College of Food Science and Engineering, Nanjing University of Finance and Economics, Nanjing 210023, China

**Keywords:** surfactin, pancreas, inflammation, gut-barrier

## Abstract

Surfactin from *Bacillus amyloliquefaciens* fmb50 was utilized to treat mice with type 2 diabetes (T2DM) induced by a high-fat diet/streptozotocin (HFD/STZ). Our group’s earlier research indicated that surfactin could lower blood glucose and mitigate liver dysfunction to further improve HFD/STZ-induced T2DM through modulating intestinal microbiota. Thus, we further investigated the effects of surfactin on the pancreas and colon in mice with T2DM to elucidate the detailed mechanism. In the present study, mice with HFD/STZ-induced T2DM had their pancreatic and colon inflammation, oxidative stress, and endoplasmic reticulum stress (ERS) reduced when given oral surfactin at a dose of 80 mg/kg body weight. According to further research, surfactin also improved glucose metabolism by activating the phosphatidylinositol kinase (PI3K)/protein kinase B (Akt) signaling pathway, further protecting islets β-cell, promoting insulin secretion, inhibiting glucagon release and mitigating pancreas dysfunction. Additionally, after surfactin treatment, the colon levels of the tight junction proteins Occludin and Claudin-1 of T2DM mice were considerably increased by 130.64% and by 36.40%, respectively. These findings revealed that surfactin not only ameliorated HFD/STZ-induced pancreas inflammation and dysfunction and preserved intestinal barrier dysfunction and gut microbiota homeostasis but also enhanced insulin sensitivity and glucose homeostasis in T2DM mice. Finally, in the further experiment, we were able to demonstrate that early surfactin intervention might delay the development of T2DM caused by HFD/STZ, according to critical biochemical parameters in serum.

## 1. Introduction

The rapidly increasing prevalence of diabetes mellitus (DM) worldwide has been one of the most serious health problems. The International Diabetes Federation reported that, in 2019, there were about 463 million adults in the world with diabetes aged 20–79 years, and the number is expected to rise to 700 million by 2045 [1]. T2DM is the most common type, accounting for 90–95% of the total diabetic population, and has become the third most common chronic disease that seriously threatens human health after tumors and cardiovascular diseases [1]. T2DM is characterized by insulin resistance (impaired responses to insulin) and β-cell dysfunction (inadequate insulin synthesis) [2,3]. Early in the disease, nutrient overload such as increase of glucose and plasma free fatty acids leads to the compensatory increase in insulin synthesis and secretion [4]. However, persistently high amounts of glucose and free fatty acids may contribute to pancreatic dysfunction through a variety of processes, including ERS, inflammation, and oxidative stress [3]. These factors in turn cause glucose intolerance and insulin resistance, which ultimately lead to T2DM [2]. Therefore, developing methods to maintain β-cell mass, preserve β-cell functions, and safeguard the pancreas could be a scientific and effective approach to mitigate T2DM [4,5].

Mounting evidence demonstrates that functional food is also regarded as a strategy for improving T2DM. Dietary polysaccharides and peptides derived from food exert anti-hyperglycaemic, anti-insulin resistance and antidiabetic effects [6]. Cinnamtannin D-1 can protect pancreatic β-cells from palmitic acid induced apoptosis by attenuating oxidative stress to further ameliorate T2DM [7]. In STZ-treated mice, fucoidan-containing food or supplements have a therapeutic effect for diabetes by preventing pancreatic β-cell damage and improving insulin synthesis [2]. By inhibiting inflammation and ERS in T2DM mice, fucoidan can also protect the pancreas and improve glucose metabolism [8]. A peptide from *Bacillus subtilis* also decreased blood glucose levels and protected β-cells from damage in diabetic rats [9]. The lipopeptide surfactin exerted an antidiabetic effect on T1DM in nonobese diabetic (NOD) mice [10]. However, few studies have concentrated on how it regulates glucose metabolism. The physiological toxicity of surfactin analysis showed that the oral medianlethaldose (LD_50_) of surfactin in mice exceeded 2500 [11] and 5000 mg/kg [12], respectively, and the acute toxicity showed that surfactin is highly safe [13,14].

STZ has been reported to be a cytotoxic chemical to the pancreatic insulin-producing β-cells of the islets of Langerhans in mammals [15]. Injection of STZ results in the degeneration of β-cells [16]. It has been discovered that utilizing STZ to induce experimental diabetes in rats is efficient, convenient, and easy to carry out [15,17]. Therefore, we investigated that the effects of surfactin on T2DM using HFD combined with STZ injection to induce T2DM mice. An earlier study conducted by our lab suggested that surfactin could migrate HFD/STZ-induced T2DM through modulating intestinal microbiota. In order to identify a potential mechanism by which surfactin may ameliorate T2DM, the effects of surfactin on inflammation and insulin signaling of the pancreas, inflammation and tight junction protein of the colon were examined in the current investigation using T2DM mice.

## 2. Results

### 2.1. Effects of Surfactin on Proteins Associated with Inflammation of the Pancreas in T2DM Mice

**Figure 1:**
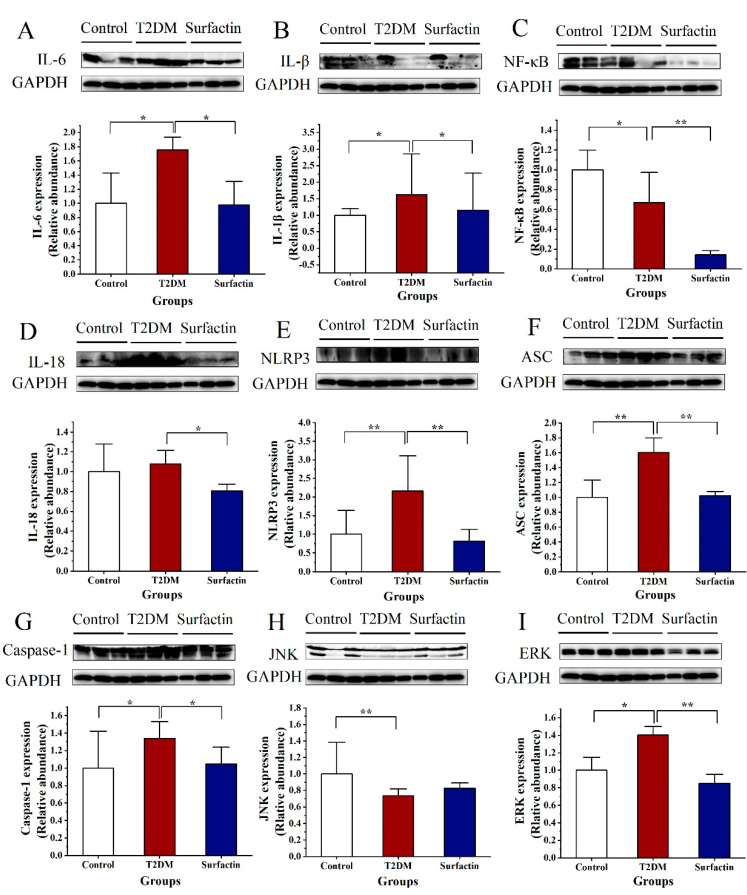
Effects of surfactin on pancreas inflammation in HFD/STZ-induced T2DM mice. The control group represents normal mice fed a basic diet, the T2DM group represents mice with T2DM induced by a 60% fat diet and 30 mg/kg body weight STZ, and the surfactant group represents T2DM mice treated with 80 mg/kg body weight surfactin. The levels of IL-6 (**A**), IL-1β (**B**), NF-κB (**C**), IL-18 (**D**), NLRP3 (**E**), ASC (**F**), Caspase-1 (**G**), JNK (**H**) and ERK (**I**). All data are presented as the means ± SD for each group. * indicates significant differences at *p* < 0.05, ** indicates significant differences at *p* < 0.01.

As shown in Figure 1A,B,E,F, the protein levels of IL-6, IL-1β, NLRP3, ASC and Caspase-1 of T2DM mice were considerably higher than those in the control mice. Compared to the T2DM mice, the expression of these protein was noticeably decreased after surfactin treatment (Figure 1). Additionally, NF-κB levels were significantly lower in T2DM mice than in the control mice, while it was significantly reduced after surfactin treatment compared to the T2DM mice (Figure 1C). Furthermore, Western blot analysis revealed that surfactin dramatically downregulated the levels of IL-18, whereas HFD/STZ treatment did not alter the protein levels of IL-18 in the T2DM mice (Figure 1D). In the present study, the protein levels of ERK in the T2DM mice were dramatically higher than the control mice, while surfactin supplementation dramatically lowered this protein level compared with the T2DM mice (Figure 1I). However, surfactin did not alter JNK protein levels in mice with HFD/STZ-induced T2DM (Figure 1H). These findings indicated that surfactin can inhibit the pancreatic inflammatory response.

**Figure 2:**
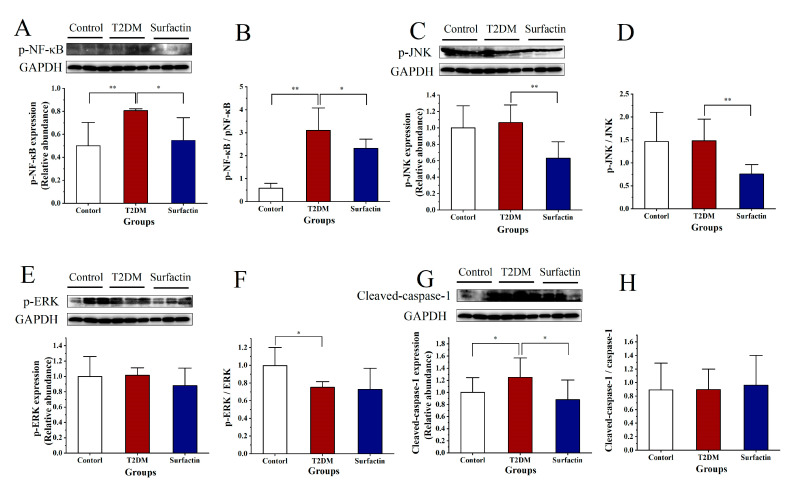
Effects of surfactin on pancreas inflammation in HFD/STZ-induced T2DM mice. The control group represents normal mice fed a basic diet, the T2DM group represents mice with T2DM induced by a 60% fat diet and 30 mg/kg body weight STZ, and the surfactant group represents T2DM mice treated with 80 mg/kg body weight surfactin. The levels of p-NF-κB (**A**), p-NF-κB/ NF-κB ratio (**B**), p-JNK (**C**), p-JNK/JNK ratio (**D**), p-ERK (**E**), p-ERK/ERK ratio (**F**), Cleaved-Caspase-1 (**G**), and Cleaved-caspase-1/caspase-1 ratio (**H**). All data are presented as the means ± SD for each group. * indicates significant differences at *p* < 0.05, ** indicates significant differences at *p* < 0.01.

As shown in Figure 2A,B, p-NF-κB levels and the p-NF-κB/ NF-κB ratio were significantly higher in T2DM mice than in the control mice, while they were significantly reduced after surfactin treatment compared to the T2DM mice. In addition, surfactin significantly reduced p-JNK protein levels and the p-JNK/JNK ratio in mice with HFD/STZ-induced T2DM (Figure 2C,D). The protein levels of cleaved-caspase-1 in the T2DM group were significantly increased compared to the control group, while surfactin treatment significantly decreased protein levels. However, the protein levels of p-ERK, the p-ERK/ERK ratio and cleaved-caspase-1/caspase-1 were not significantly different among the three groups (Figure 2E–H). These findings indicated that surfactin can inhibit the pancreatic inflammatory response. In addition, more sample results are shown by Western blot analysis in the Figure S1. 

### 2.2. Effects of Surfactin on the Pancreatic Histopathology and Function in T2DM Mice

**Figure 3:**
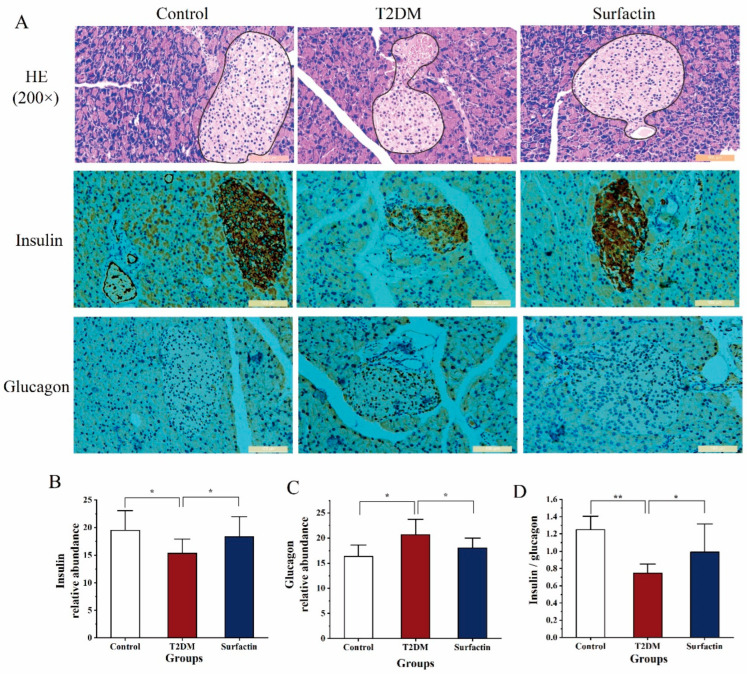
Effects of surfactin on pancreas tissue and function in HFD/STZ-induced T2DM mice. The control group represents normal mice fed a basic diet, the T2DM group represents mice with T2DM induced by a 60% fat diet and 30 mg/kg body weight STZ, and the surfactin group represents T2DM mice treated with 80 mg/kg body weight surfactin. H&E staining of the pancreas and immunohistochemistry for insulin and glucagon (**A**), the corresponding relative abundance of insulin (**B**), the corresponding relative abundance of glucagon (**C**) and the ratio of insulin to glucagon (**D**). * indicates significant differences at *p* < 0.05, ** indicates significant differences at *p* < 0.01.

According to morphometric analysis (Figure 3A), the pancreatic islets of normal mice were mostly round or oval with clear boundaries and regular arrangement. In the T2DM mice, STZ induced compensatory hypertrophy of pancreatic cells, islets showed irregular contours and caused the cell boundary to blur or disappear [18], our results also confirmed this conclusion However, these phenomena were obviously reversed after surfactin treatment. An apparent increase in the size and number of islets was noticed in the T2DM mice after surfactin treatment. These results showed that surfactin effectively reduced the pancreas damage.

An impaired pancreas can lead to dysfunction of islet cells, thus affecting the normal secretion of insulin and glucagon. In the present study, STZ exposure significantly inhibited islet β-cells’ ability to secrete insulin and reduced the ratio of insulin to glucagon compared to the control mice. However, these parameters were significantly reversed after surfactin supplementation compared to the T2DM mice (Figure 3B,D). In addition, surfactin treatment noticeably inhibited higher levels of glucagon release following STZ exposure (Figure 3C); this is similar to the control mice. These suggested that surfactin improved pancreas function.

### 2.3. Effects of Surfactin on Proteins Associated with Glycometabolism of the Pancreas in T2DM Mice

**Figure 4:**
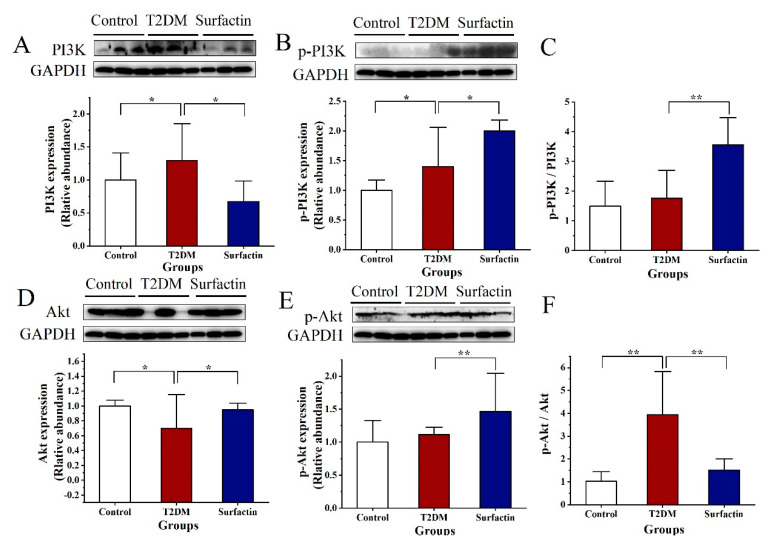
Effects of surfactin on proteins associated with glycometabolism of the pancreas in HFD/STZ-induced T2DM mice. The control group represents normal mice fed a basic diet, the T2DM group represents mice with T2DM induced by a 60% fat diet and 30 mg/kg body weight STZ, and the surfactin group represents T2DM mice treated with 80 mg/kg body weight surfactin. The levels of PI3K (**A**), p−PI3K (**B**), the ratio of p−PI3K to PI3K (**C**), Akt (**D**), p−Akt (**E**) and the ratio of p−Akt to Akt (**F**). * indicates significant differences at *p* < 0.05, ** indicates significant differences at *p* < 0.01.

In the present study, PI3K protein levels in T2DM mice were significantly higher than the control mice, while surfactin significantly downregulated this protein expression compared with the T2DM mice (Figure 4A), and the trend is similar to the normal mice. After surfactin treatment, the protein levels of p-PI3K and the ratio of p-PI3K to PI3K were noticeably increased compared to that in the T2DM mice (Figure 4B,C). T2DM mice had much lower levels of the protein Akt than the control mice, whereas T2DM mice treated with surfactin had significantly higher levels of Akt than the T2DM mice (Figure 4D). In comparison to the T2DM mice, surfactin also noticeably increased the protein levels of p-Akt (Figure 4E). Conversely, the ratio of p-Akt to Akt was highly increased in T2DM mice compared to the control mice, but it was significantly downregulated in T2DM mice following surfactin treatment (Figure 4F), and the trend is closer to the normal mice. In addition, the raw data for pancreas protein were shown in the Table S1.

### 2.4. Effects of Surfactin on Proteins Associated with Inflammation of the Colon in T2DM MICE

**Figure 5:**
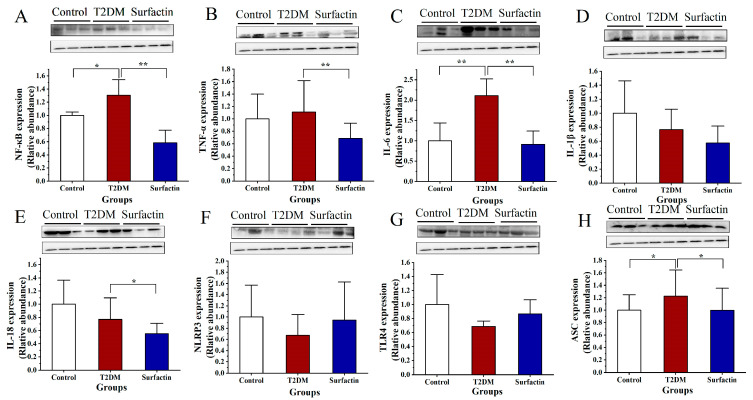
Effect of surfactin on colon inflammation in HFD/STZ-induced T2DM mice. The control group represents normal mice fed a basic diet, the T2DM group represents mice with T2DM induced by a 60% fat diet and 30 mg/kg body weight STZ, and the surfactin group represents T2DM mice treated with 80 mg/kg body weight surfactin. The levels of NF-κB (**A**), TNF-α (**B**), IL-6 (**C**), IL-1β (**D**), IL-18 (**E**), NLRP3 (**F**), TLR4 (**G**) and ASC (**H**). * indicates significant differences at *p* < 0.05, ** indicates significant differences at *p* < 0.01.

**Figure 6:**
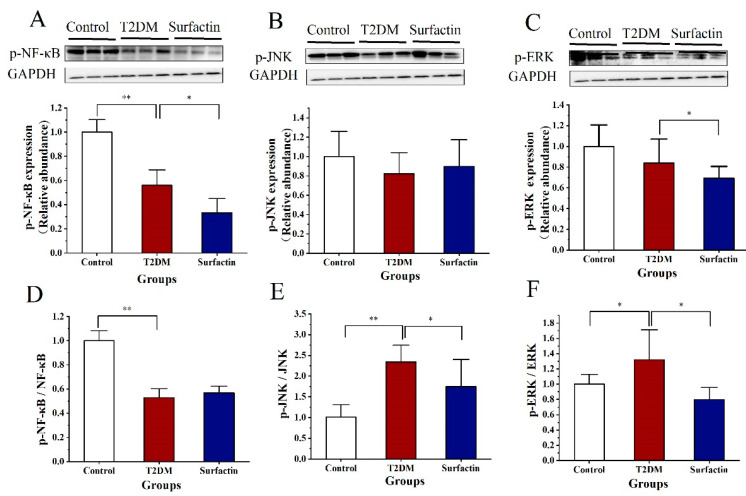
Effect of surfactin on colon inflammation in HFD/STZ-induced T2DM mice. The control group represents normal mice fed a basic diet, the T2DM group represents mice with T2DM induced by a 60% fat diet and 30 mg/kg body weight STZ, and the surfactin group represents T2DM mice treated with 80 mg/kg body weight surfactin. The levels of p-NF-κB (**A**), p-JNK (**B**), p-ERK (**C**), p-NF-κB/NF-κB (**D**), p-JNK/JNK (**E**) and p-ERK/ERK (**F**). * indicates significant differences at *p* < 0.05, ** indicates significant differences at *p* < 0.01.

As shown in Figure 5A,C,H, HFD/STZ substantially increased the protein levels of NF-κB, IL-6 and ASC in comparison to the control group, while surfactin treatment noticeably decreased these proteins in comparison to the T2DM group. The protein levels of TNF-α and IL-18 were not significantly different between the control group and the T2DM group. However, surfactin supplementation noticeably inhibited these proteins’ expression (Figure 5B,E). The protein levels of TGF-β of the T2DM mice were significantly suppressed compared with that in the control group, while surfactin treatment also significantly promoted TGF-β expression compared with that in the T2DM group (Figure 5C). JNK protein levels in the T2DM mice in the present study were not significantly different from those in the control mice, but surfactin supplementation dramatically reduced their levels as compared to the T2DM mice (Figure 5D). In addition, Western blot analysis revealed that surfactin did not alter the protein levels of IL-1β, NLRP3, TLR4, and ERK in mice with HFD/STZ-induced T2DM (Figure 5D,F,G and Figure 6E). These results revealed that surfactin also inhibited the colonic inflammatory response. Additionally, the raw data for colon protein were shown in the Table S2.

As shown in Figure 6A,D, HFD/STZ substantially decreased the protein levels of the p-NF-κB in comparison to the control group, while surfactin treatment further noticeably decreased these proteins but did not alter p-NF-κB/NF-κB ratio in comparison to the T2DM group. p-JNK protein levels in the T2DM mice in the present study were not significantly different from those in the surfactin group (Figure 6B). In addition, the p-JNK/JNK ratio was significantly higher than the control group, while surfactin treatment significantly lowered this ratio compared to the T2DM group (Figure 6E). Surfactin treatment also significantly downregulated the levels of p-ERK and the p-ERK/ERK ratio compared to the T2DM mice (Figure 6C,F). These results revealed that surfactin also inhibited the colonic inflammatory response. The protein levels of Caspase-1 were not significantly different between the control group and the T2DM group. However, surfactin supplementation noticeably inhibited these proteins’ expression (Figure S2A). Interestingly, the protein levels of Cleaved-caspase-1 of the T2DM mice were significantly suppressed compared with that in the control group, while surfactin treatment significantly increased the Cleaved-caspase-1/caspase-1 ratio but did not alter Cleaved-caspase-1 levels compared with that in the T2DM group; this trend is similar to the control group (Figure S2B,C).

### 2.5. Effects of Surfactin on the Colonic Tight Junction Protein in T2DM Mice

**Figure 7:**
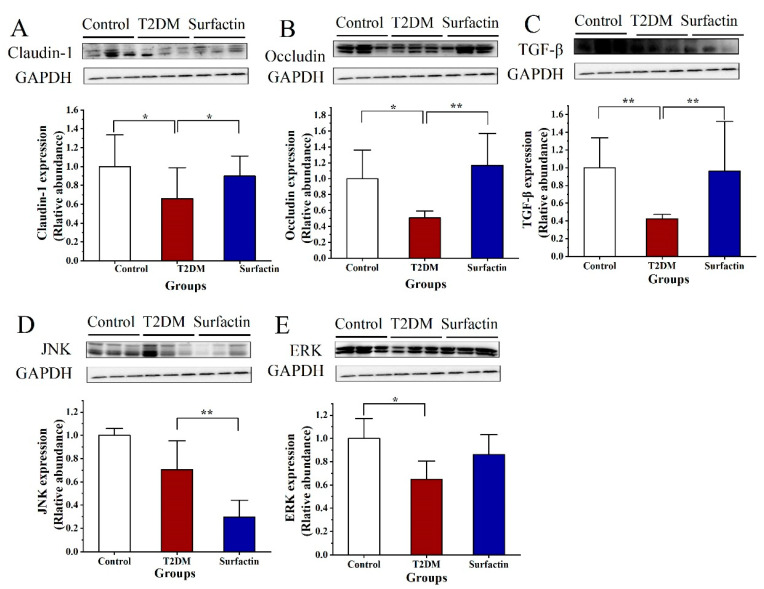
Effect of surfactin on critical protein involved intestinal barrier function and stress in HFD/STZ-induced T2DM mice. The control group represents normal mice fed a basic diet, the T2DM group represents mice with T2DM induced by a 60% fat diet and 30 mg/kg body weight STZ, and the surfactin group represents T2DM mice treated with 80 mg/kg body weight surfactin. The levels of Claudin-1 (**A**), Occludin (**B**), TGF-β (**C**), JNK (**D**) and ERK (**E**). * indicates significant differences at *p* < 0.05, ** indicates significant differences at *p* < 0.01.

The protein levels of Claudin-1 and Occludin of the T2DM mice in the colon were noticeably downregulated compared with that in the control group, while these levels in the T2DM group were noticeably upregulated after surfactin supplementation (*p* < 0.05) (Figure 7A,B). 

### 2.6. Effects of Surfactin on Glucose Metabolism-Related Parameters of the Serum in T2DM Mice

**Figure 8:**
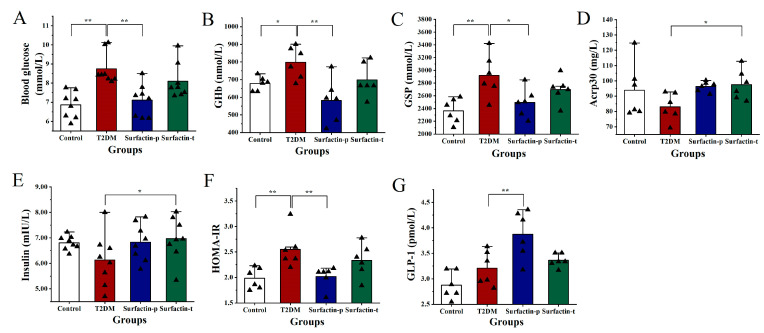
Effect of surfactin on critical serum parameters in HFD/STZ-induced T2DM mice. The control group represents normal mice fed a basic diet, the T2DM group represents mice with T2DM induced by a 60% fat diet and 30 mg/kg body weight STZ, the surfactin-p group represents T2DM mice treated with 80 mg/kg body weight surfactin before STZ injection, and the surfactin-t group represents T2DM mice treated with 80 mg/kg body weight surfactin after STZ injection. The serum levels of blood glucose (**A**), GHb (**B**), GSP (**C**), Acrp30 (**D**), insulin (**E**), HOMA-IR (**F**) and GLP-1 (**G**). * indicates significant differences at *p* < 0.05, ** indicates significant differences at *p* < 0.01.

To confirm the effects of surfactin on mice with HFD/STZ-induced T2DM, serum parameters involved in glucose metabolism were measured. The surfactin-p group represents T2DM mice treated with 80 mg/kg body weight surfactin before STZ injection, the surfactin-t group represents T2DM mice treated with 80 mg/kg body weight surfactin after STZ injection, and the other grouping is the same as mentioned above. Figure 8 shows that the serum levels of glucose, GHb, GSP and HOMA-IR of T2DM mice were noticeably higher than the control mice, while after surfactin treatment, the levels of these parameters in the surfactin-p group were noticeably lower compared with the T2DM mice. However, these parameters were not significantly different between the T2DM group and the surfactin-t group. Surfactin also significantly promoted GLP-1 production in the surfactin-p group compared with that in the T2DM group (Figure 8G). In addition, the surfactin-t group’s serum levels of Acrp30 and insulin were significantly higher than the T2DM mice, whereas the HFD/STZ treatment had no effect on these parameters in comparison to the control mice (Figure 8D,E). In addition, raw data were shown in the Table S3, and inflammation and antioxidant-related indicators are shown in Figures S3 and S4.

## 3. Discussion

Some studies have revealed an inflammatory process in the pancreas of patients with T2DM characterized by the presence of immune cells, cytokines, and β-cell apoptosis [19]. Both cytokine-induced β-cell death and pancreas dysfunction need activation of the transcription factor NF-κB [20]. Inflammatory cells are activated, exhibit direct cytotoxicity, or release pro-inflammatory cytokines such as IL-1β and IL-6, which are crucial in the beginning of a number of pathological processes in pancreatitis. These pro-inflammatory cytokines then trigger inflammatory cascades, leading to a systemic inflammatory response and tissue dysfunction [21]. The present study found that surfactin treatment significantly decreased pancreas levels of NF-κB, p-NF-κB, IL-1β, IL-18 and IL-6, indicating that surfactin partially inhibited the inflammatory response in the pancreas by inactivating the NF-κB signaling pathway. The other pathway involved in pancreatitis is mediated through MAPKs. Two MAPK subfamilies, p38 and ERK1/2, have recently been demonstrated to be essential in the process driving cytokine production in pancreatitis [22]. According to the Western blot in this investigation, surfactin decreased NF-κB, p-NF-κB and ERK levels, but did not alter p-ERK levels and the p-ERK/ERK ratio in the pancreas. We hypothesized that surfactin inhibited pancreas inflammations and dysfunction via the inhibition of the inflammatory response due to the inactivation of the NF-κB signal pathways in T2DM mice.

Defective insulin secretion and reactions in T2DM are caused by multiple mechanisms. Glucotoxicity, oxidative stress and ERS all contribute to the dysfunction of the islets [23]. Elevated glucose concentrations in pancreatic islets cause the islet cells to become more metabolically active, which increases the formation of ROS. ROS then promote the activation of the NLRP3 inflammasome and Caspase-1, which allows the production of mature IL-1β [24]. Increased insulin demand and production induces ERS, which also activates the inflammasome [25]. In addition, several studies using genetically modified mice that lack inflammasome components NLRP3, ASC, and Caspase-1 provided initial evidence that activation of the NLRP3 inflammasome is a key mechanism that induces metabolic inflammation and insulin resistance [26]. Deactivation of the NLRP3 inflammasome is accompanied by improved glucose homeostasis in obese T2DM patients who lose excess weight through dietary intervention, suggesting that inflammasome may be a clinically significant mechanism linking inflammation with T2DM [26]. According to Lee et al. (2013) [27], monocytes from newly discovered untreated T2DM patients have increased expression of the inflammasome’s NLRP3 and ASC, as well as enhanced Caspase-1 activation. In the present study, the pancreas levels of IL-1β, NLRP3, ASC, Caspase-1, Cleaved-caspase-1, JNK and p-JNK of HFD/STZ-induced T2DM mice were significantly higher than the control mice, while surfactin supplementation significantly reversed their expressions. This reveals that the deactivation of the NLRP3 inflammasome by surfactin may reduce pancreatic stress and IL-1β production. The JNK pathway has been identified as an underlying molecular mechanism of β-cell deterioration by oxidative stress, its activation is involved in the reduction of both insulin gene expression and secretion [28]. After TNF-α stimulation, the JNK signaling suppressors may improve insulin action and avoid glucose metabolism abnormalities [29]. In the present study, the pancreas levels of JNK were significantly decreased in HFD/STZ-induced T2DM mice after surfactin treatment. Surfactin suppressed oxidative stress, ERS (which is caused by inactivating the NLRP3 inflammasome) and inhibited JNK signaling together to reduce pancreas dysfunction and insulin action.

A complete structure is the basis of normal physiological function of islets. T2DM’s incidence and progression are increasingly influenced by β-cell dysfunction, which can also cause increased insulin resistance and damage to the islet microstructure [30]. Chronic hyperglycaemia causes β-cell dysfunction through the activation of the JNK kinase pathway and the suppression of the PI3K/Akt pathway [12]. However, chronic long-term hyperglycaemia can cause β-cells to pathologically decompensate and fail to meet the insulin demand, it further in turn leads to β-cell dysfunction and the development of T2DM [2]. Insulin secreted by islet β-cells not only interferes with the signaling pathway of PI3K/Akt in target organs and tissues, but also interposes the signaling pathway of PI3K/Akt by the pancreas itself, hence affecting the islet β-cell function and the glucose homeostasis [7]. Increased inflammatory cytokines and persistent ERS during the stress state might activate aberrant JNK phosphorylation, which then results in IRS serine phosphorylation, further suppressing the downstream PI3K/Akt pathway and blocking insulin signal transduction [8,31]. However, surfactin treatment significantly upregulated the pancreas levels of p-PI3K, Akt and p-Akt and downregulated JNK levels in HFD/STZ-induced T2DM mice. This finding suggests that surfactin may alleviate pancreas islet β-cells’ dysfunction via inhibiting the JNK signaling pathway to activate the PI3K/Akt signaling pathway. The islets, on the other hand, respond to the increased insulin demand in the prediabetic, insulin-resistant state by enhanced insulin secretion and increased β-cell mass to generate compensatory hyperinsulinemia and maintain relative euglycemia [8]. However, when T2DM emerges, β-cell function and mass are significantly decreased, and there is insufficient insulin secretion to compensate for the insulin resistance, leading to the development of the chronic hyperglycaemic diabetic state [32]. In the present study, mice with HFD/STZ-induced T2DM also had insulin resistance and decreased β-cell function and mass. However, treatment with surfactin significantly reversed these symptoms, suggesting that surfactin protected the pancreas from tissue damage and dysfunction.

Systemic inflammation is regarded as the main cause of insulin resistance in individuals with T2DM, including colon inflammation. Initiation and perpetuation of intestinal inflammation are mostly mediated by the proinflammatory cytokines IL-1β and IL-6 [33]. TNF-α augments proinflammatory cytokines by macrophages and T cells, which further alters the barrier and results in the death of intestinal epithelial cells [34]. All proinflammatory cytokine genes contain NF-κB binding sites, and their transcription is controlled by these factors [35]. In the present study, mice induced by HFD/STZ had markedly higher colon levels of IL-18, IL-6, TNF-α, NF-κB, and p-NF-κB, whereas surfactin significantly lowered their expression. NLRP3 inflammasome can initiate and activate signaling via activating NF-κB signaling and stimulate the body to produce inflammatory cytokines. ASC levels in the colon were markedly increased by HFD/STZ treatment, but this improvement was inhibited after surfactin supplementation. In addition, there were no significant differences in the colon levels of NLRP3 and TLR4 among the three groups. Therefore, we hypothesize that surfactin not only directly restrains the inflammatory factors produced by NF-κB activation, but also inhibits NF-κB initial activation by inhibiting ASC expression of the NLRP3 inflammasome. The levels of JNK and ERK associated with oxidative stress and ERS were significantly higher than the control mice, while surfactin treatment significantly lowered JNK expression, p-JNK / JNK ratio, p-ERK expression and p-ERK/ERK ratio, but not ERK expression, compared with the T2DM mice. This also indirectly contributes to the reduction of intestinal inflammation. In addition, the colon levels of Caspase-1 were not significantly different between the T2DM group and the control group, while surfactin significantly decreased the colon levels of Caspase-1 compared to the T2DM group. However, surfactin did not alter the colon levels of Cleaved-caspase-1 compared to the T2DM group. Collectively, our findings revealed that surfactin protects gut barrier function, probably by inactivating NF-κB and NLRP3 inflammatory signaling and lowering oxidative stress and ERS. TNF-α and IL-1β can also alter the expression and distribution of Occludin and Claudin-1, which are associated with epithelial barrier function via cell signaling pathways [36], further increasing the permeability of gut barrier. In the present study, HFD/STZ-induced T2DM mice exhibited an impaired intestinal barrier as well as downregulated Occludin and Claudin-1. Western blot analysis revealed that all symptoms had greatly improved after surfactin treatment. Additionally, TGF-β was a prominent anti-inflammatory cytokine that was dramatically decreased in T2DM mice compared to the control mice, while surfactin treatment significantly increased its levels; this trend was similar to the control mice. In short, surfactin mitigated gut-barrier dysfunction due to the inhibition of inflammation and oxidative stress and ERS. 

Recent evidence strongly demonstrates an inextricable link between the pancreas and the gut. A growing body of evidence has emerged to support that the pancreatic exocrine function affects the gut immunity, which further supports the pancreas–intestinal axis [37]. Pathobionts invading the epithelium can translocate to underlying layers and be disseminated to other organs (e.g., to the mesenteric LN via lymphatics or liver via the portal vein, and the pancreatic duct en route to the pancreas) [38]. Translocations trigger innate and adaptive immune responses and induce inflammation of colonized tissues or organs [39]. Whenever the intestinal barrier is disrupted by inflammation, the risk of bacterial translocation is increased. Comparatively to the HFD/STZ-induced T2DM mice, surfactin treatment in the present study significantly increased the colon levels of Occludin and Claudin-1, further reducing intestinal permeability. This contributed to inhibited bacterial translocation. In addition, *Bifidobacterium*, a popular intestinal probiotic, can reduce the gastrointestinal dysfunction [40]. *Bacteroides* enhance the differentiation of goblet cells, leading to an increase in the number of goblet cells and mucin gene expression in the colon of gnotobiotic rats [41]. It has been discovered that *Prevotella* is essential for maintaining the gut barrier and ameliorating intestinal inflammation. However, the relative abundance of *Bifidobacterium, Bacteroides* and unidentified *Prevotella* of T2DM mice were noticeably increased after surfactin intervention compared to the T2DM mice [42]. According to the previous finding, we hypothesized that surfactin improved gut barrier function by increasing *Bifidobacterium*, *Bacteroides* and unidentified *Prevotella* to alleviate intestinal inflammation. The effect of *Akkermansia* on barrier integrity has been contradictory in previous studies. *Akkermansia*-induced mucus degradation may stimulate renewal, thereby improving barrier function, and it also facilitates dextran sulfate sodium (DSS)-induced intestinal inflammation in mice [43,44]. However, the mice’s colonic length was noticeably reduced by *A. muciniphila* in acute colitis, and the mice’s daily disease activity index (DAI) score was higher than in the DSS-induced mice after *A. muciniphila* treatment [45]. The T2DM mice showed a higher relative abundance of *Akkemansia*, but early surfactin intervention reversed this pattern, suggesting that *Akkemansia* may be essential for maintaining the integrity of the intestinal barrier [42]. Increasing evidence suggests that the pancreas may play a role in defending against invader pathobionts and maintaining the balance of the intestinal flora. The pancreatic exocrine function has been shown to contribute significantly more to the composition of intestinal microorganisms than any other host factor in individuals without pancreatic disease [46]. Local homeostasis is disturbed when gut bacteria translocate into the pancreas. An *E. coli* MG1655 mono-colonized pancreatitis rat model demonstrated more severe pancreatic injury as compared to a normal pancreatitis rat model with significant upregulation of the TLR-4-mitogen-activated protein pathway and activation of the ERS pathway in intestinal epithelial cells [40]. NLPR3 may be a crucial factor in the gut microbiota-pancreatitis axis [47]. However, surfactin treatment inactivated the pancreatic NLRP3 signaling pathway but had no effect on the TLR4 pathway in the T2DM mice. In the pancreas–intestinal axis, surfactin mitigated colonic inflammation and dysfunction by modulating gut microbiota and preserving gut barrier function, further inhibiting pancreas damage and improving pancreatic dysfunction.

In the further research, serum levels of GHb and GSP, which are key indicators of long-term blood glucose, were significantly decreased after surfactin treatment in the surfactin-p group. Acrp30, an insulin-sensitizing hormone, has shown anti-inflammatory and anti-diabetic potential in clinical trials and can alleviate insulin resistance in mice [8]. However, in the present study, serum levels of Acrp30 in the surfactin-p group significantly increased after surfactin treatment compared to the T2DM mice. After surfactin treatment, HOMA-IR levels were also noticeably lowered, suggesting that surfactin intervention improved insulin resistance and hyperglycemia in HFD/STZ-induced T2DM mice. GLP-1 is capable of stimulating insulin secretion from pancreatic β-cells, and suppressing glucagon release [2], it also prevents pancreatic β-cell mass loss under diabetic conditions by inhibiting the apoptosis and enhancing the proliferation of β-cells via binding to its GLP-1 receptor (GLP-1R) existing on pancreatic β-cells [48]. Activation of ERK1/2 and PI3K/Akt signaling has been considered as a major mechanism accounting for the beneficial effects of GLP-1R [3]. We observed that surfactin intervention significantly increased GLP-1 levels; this may have contributed to lower blood glucose of T2DM mice. After early surfactin intervention, lower serum levels of proinflammatory cytokines (such as TNF-α, TNF-α and IL-1β), MDA and improved gut microbiota balance were also observed (Supplementation File). After STZ injection, to treatment T2DM mice by oral surfactin, these parameters were slightly reversed compared to the T2DM mice. Collectively, we confirmed early surfactin intervention is critical for mitigating HFD/STZ-induced T2DM in mice.

## 4. Materials and Methods

### 4.1. Materials and Reagents

The *Bacillus amyloliquefaciens* fmb50 strain was obtained from the laboratory of Enzyme Engineering at Nanjing Agricultural University’s College of Food Science and Technology (Nanjing, China) [13,14]. Enzyme-linked immunosorbent assay (ELISA) kits for Glucagon-like peptide-1 (GLP-1), glycated haemoglobin (GHb), glycated serum protein (GSP) and adiponectin (Acrp30) and insulin were purchased from Jiangsu Meimian Industry Co., Ltd. (Yancheng, Jiangsu, China). STZ (Cas: 18883-66-4) was purchased from Shanghai Aladdin Biochemical Technology Co., Ltd. (Shanghai, China). The primary antibodies against tumor necrosis factor-α (TNF-α), nuclear factor kappa-B (NF-κB), interleukin-1β (IL-1β), IL-6, IL-18, transforming growth factor-β (TGF-β), PI3K, phosphorylated-PI3K (p-PI3K), Akt, phosphorylated- Akt (p-Akt), ASC, toll-like receptor 4 (TLR4), NOD-like receptor thermal protein domain associated protein 3 (NLRP3), cysteinyl aspartate specific proteinase-1 (Caspase-1), c-Jun N-terminal kinase (JNK), ERK, zonula occludens-1 (ZO-1), Claudin-1, Occludin and glyceraldehyde-3-phosphate dehydrogenase (GAPDH) were purchased from Beyotime Institute of Biotechnology, (Shanghai, China). Phosphorylated-caspase-1(p-caspase-1), phosphorylated-JNK (p-JNK), phosphorylated-ERK (p-ERK) and phosphorylated-NF-κB (p-NF-κB) were purchased from Cell Signaling Technology Co., Ltd. (Boston, MA, USA). All other analytical reagents were purchased from Sinopharm Chemical Reagent Co., Ltd. (Shanghai, China). All other analytical reagents were purchased Sinopharm Chemical Reagent Co., Ltd. (Shanghai, China).

### 4.2. Fermentation and Production of Surfactin from Bacillus Amyloliquefaciens fmb50

Surfactin samples were obtained from fermentation broths and then flocculated with chitosan (0.5 g/L), and sodium alginate (0.3 g/L), pH = 5.0, before being dissolved in 100% ethanol and freeze-dried. High performance liquid chromatography (HPLC) (U-3000, Dionex, Sunnyvale, CA, USA) was carried out equipped with an Agilent C18 column (4.5 mm × 250 mm, Agilent, Palo Alto, CA, USA) and a UV detector was used to identify the surfactin sample [49]. In brief, the surfactin sample was injected into the column and then eluted with acetonitrile with 0.1% TFA at a flow rate of 0.84 mL/min. Eluent absorbance was monitored at 210 nm. The purity of surfactin was approximately 88.6% (the result is shown in the Figure S5) after purification [13], and the collected surfactin dry powder was stored at 4 °C for following tests.

### 4.3. Animal Experimental Design

A total of thirty healthy 4 week-old male Kunming mice were provided by Beijing Vital River Laboratory Animal Technology Co., Ltd. (Beijing, China). The production license number of the experimental animals is SCXK (Jiansu) 2012-0004. All experiments were approved by the Ethics Committee and conducted according to the Guide for the Care and Use of Laboratory Animals (permit NO. NJAU. No20210317024). Feeding environment and treatment refer to previous studies [13]. Subsequently, mice were randomly divided into three groups, each containing ten mice. In detail, each group was divided to 2 sub-groups of 5 mice each. The control group was fed with a basic diet (Xietong Co., Nanjing, China), while the T2DM group and surfactin group were fed with a 60% fat diet (Xietong CO., Nanjing, China) for 12 weeks. The surfactin group were administrated with surfactin of 80 mg/kg body weight.

The fasting blood glucose (FBG) was assessed before induction with STZ, after 4 weeks. The mice in the T2DM and surfactin group received two intraperitoneal injections of 30 mg/kg·body weight STZ at intervals of one week [50]. The control group were injected with equivalent volume of citrate buffer (citric acid, Cas, 5949-29-1: sodium citrate, Cas, 68-04-2, 1:1). FBG of all mice was measured by tail tip after STZ injection for three days. The mice with FBG ≥ 7.8 mmol/L were marked as T2DM animals [50].

In addition, the control group and T2DM group were kept the same as the preceding experiment, but we redesigned the group to verify the amelioration effect of surfactin on T2DM (prevention or treat). Specially, the surfactin prevention group (surfactin-p) received 80 mg/kg of surfactin before the STZ injection, while the surfactin treat group (surfactin-t) received 80 mg/kg of surfactin after the STZ injection. The other schedule followed the same protocol as the above experiment.

### 4.4. Collection and Preparation of Samples

All of the mice were humanely sacrificed at the end of experiment to collect their pancreas and colon tissue. The remaining tissues were kept at −80 °C for further analysis, while some of the tissue was fixed in 4% neutral formaldehyde solution for histological examination. The serum was collected after 3500 rpm centrifugation at 4 °C for 15 min and stored at −80 °C for subsequent analysis.

### 4.5. Measurement for Critical Parameters in Serum

Using ELISA kits in accordance with the manufacturer’s instructions, the serum levels of GLP-1, GHb, GSP, Acrp30 and insulin were measured. HOMA-IR was calculated according to the formula: HOMA-IR = FBG (mmol/L) × FINS (mIU/L)/22.5. The serum glucose levels were also determined.

### 4.6. Histological Analysis

The pancreas was fixed in a 4% neutral formaldehyde solution before being tissue-embedded in paraffin. The tissue sections were the divided into 4 μm thick sections, some of which were used for hematoxylin-eosin (HE) staining to assess organic alteration. Additionally, immunohistochemical staining was performed to assess the pancreas’s secretion of insulin and glucagon. Photomicrographs were captured with a light microscope equipped with a camera (Nikon Eclipse 80i, Nikon Co., Tokyo, Japan). Image J software was used to analyze the image’s relative fluorescence intensity.

### 4.7. Western Blot Analysis

Total proteins in the colon and pancreas were isolated using radioimmunoprecipitation assay (RIPA) buffer with 1 mM phenylmethylsulfonyl fluoride (PMSF) (Beyotime Institute of Biotechnology, Shanghai, China). Western blot analysis was performed using the supernatant of whole-tissue lysates, which was collected by centrifugation at 14,000× *g* for 5 min at 4 °C (protein electrophoresis instrument, Yeasen Biotech Co., Ltd., Shanghai, China). The proteins were transferred to the nitrocellulose (NC) membrane (GE Healthcare Life Science, Pittsburgh, PA, USA) after being separated using 6% and 12% sodium dodecyl sulfate–polyacrylamide gels (SDS-PAGE, Vazyme Biotech. Co., Ltd., Nanjing, China; GoldBand 3-color Regular Range Protein Marker, Yeasen Biotech Co., Ltd., Shanghai, China). The membranes were incubated with diluted primary rabbit antibodies (the dilutions for the target protein: 1:1000–2000; the dilutions for GAPDH: 1:10,000) at 4 °C overnight after blocking with 5% skim dry milk for 2 h. The membranes were incubated with HRP-linked secondary antibody anti-rabbit IgG (Beyotime Institute of Biotechnology, Shanghai, China) for 2 h after being washed three times with TBST solution (TBST, Beijing Solarbio Science & Technology Co., Ltd., Beijing, China). The bands were then scanned with the ECL plus solution (Beyotime Institute of Biotechnology, Shanghai, China). The internal standard was considered to be GAPDH. Image J software was used to quantify the target band density.

### 4.8. Statistical Analysis

The data are presented as the means ± standard derivations (SD). One-way analysis of variance (ANOVA) with (SPSS statistics software, Chicago, IL, USA) was used to analyze differences between the groups, followed by Duncan’s test for multiple comparisons. *p* < 0.05 and *p* < 0.01 indicated significant differences.

## 5. Conclusions

**Figure 9:**
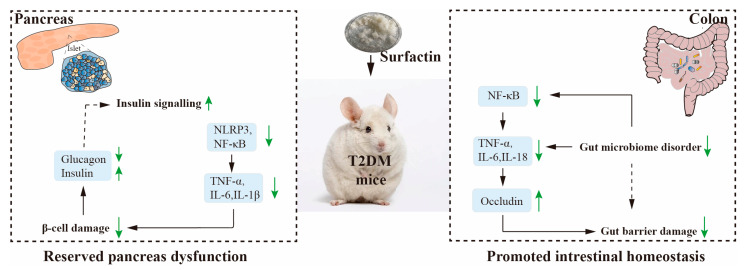
Potential mechanism by which surfactin alleviates T2DM. Green signifies (up) increased parameters, metabolites, or proteins, green signifies (down) decreased parameters, metabolites, or proteins in the surfactin group.

By repairing the impaired pancreas and improving insulin resistance, we were able to show that oral administration of surfactin reversed metabolic dysfunctions and inflammation for T2DM treatment in HFD/STZ-induced T2DM mice. Surfactin was thought to prevent T2DM by, first, reversing pancreatic dysfunctions through reducing oxidative stress, ERS and inflammatory responses, and second, improving insulin resistance by regulating the PI3K/AKT signaling pathway. Surfactin also had a beneficial impact on the gut barrier by inhibiting gut inflammation and boosting tight junction protein in the T2DM mice (Figure 9). In conclusion, surfactin may become a viable option for T2DM prevention.

## Data Availability

The data presented in this study are available in [Surfactin Mitigates a High-Fat Diet and Streptozotocin-Induced Type 2 Diabetes through Improving Pancreatic Dysfunction and Inhibiting Inflammatory Response or supplementary material Figures S1–S5 and Tables S1–S3].

## Supplementary Materials

The supporting information can be downloaded at: https://www.mdpi.com/article/10.3390/ijms231911086/s1.
