# Supplementary material for: Surfactin Mitigates a High-Fat Diet and Streptozotocin-Induced Type 2 Diabetes through Improving Pancreatic Dysfunction and Inhibiting Inflammatory Response
Source: Int J Mol Sci. 2022 Sep 21;23(19):11086. doi: 10.3390/ijms231911086 (PMC9570334; doi:10.3390/ijms231911086)
Supplement: Supplementary file 1 [file ijms-23-11086-s001.zip › ijms-1887285-supplementary.pdf]

1. Results

1.1 Effects of surfactin on related inflammatory responses of the serum in T2DM mice

The serum levels of IL-1β in the T2DM group were significantly increased compared to the control group, while the levels of IL-1β in the surfactin-p group were significantly decreased than the T2DM group (Figure S3A). The levels of IL-1β in the surfactin-t group were not altered after HFD/STZ exposure (Figure S3A). The levels of TNF-α of the T2DM mice were noticeably higher than the control mice, while surfactin (in the surfactin-p and surfactin-t group) treatment noticeably lower this cytokine compared to the T2DM mice (Figure S3B). HFD/STZ treatment did not significantly alter the serum levels of NF-κB (Figure S3D). However, surfactin treatment (in the surfactin-t and surfactin-t group) significantly reduced the levels of NF-κB. In addition, surfactin supplementation did not alter the levels of IL-6 after HFD/STZ treatment (Figure S3C).

2.2. Effects of surfactin on related anti-oxidation of the serum in T2DM mice

The serum levels of MDA of the T2DM mice were significantly increased than the control mice, while the levels of MDA were significantly decreased after surfactin treatment (in the surfactin-t and surfactin-t group) (Figure S4D). The serum activities of CAT in the surfactin-t group were dramatically enhanced compared to the T2DM group (Figure S4A). In addition, surfactin treatment did not alter SOD and GSH-Px activities compared to the T2DM mice (Figure S4B-C).

2.3 High performance liquid chromatography data

Using the standard substance of surfactin, a standard curve was drawn with the concentration of surfctin as the abscissa and the peak area as the ordinate (Figure. S5). The formula for calculating the purity of surfactin samples is: measured concentration * total volume / total sample weight. The concentration of surfactin sample 1-3 calculated according to the formula is as follows: 90.58%,

88.82, 86.68% (Figure S5). Therefore, the purity of surfactin was approximately 88.6%.

FIGURE CAPTIONS

Figure S3. Effects of surfactin on serum inflammatory cytokines in HFD/STZ-induced T2DM mice. The control group represents normal mice fed a basic diet, the T2DM group represents mice with T2DM induced by a 60% fat diet and 30 mg/kg body weight STZ, and the surfactin-p group represents T2DM mice treated with 80 mg/kg body weight surfactin before STZ injection, the surfactin-p group represents T2DM mice treated with 80 mg/kg body weight surfactin after STZ injection. The levels of IL-1β (A), TNF-α (E), IL-6 (F) and NF-κB (D). * indicate significant differences at $p < 0.05$, ** indicate significant differences at $p < 0.01$.

Figure S4. Effects of surfactin on serum parameters associated with antioxidant in HFD/STZ-induced T2DM mice. The control group represents normal mice fed a basic diet, the T2DM group represents mice with T2DM induced by a 60% fat diet and 30 mg/kg body weight STZ, and the surfactin-p group represents T2DM mice treated with 80 mg/kg body weight surfactin before STZ injection, the surfactin-p group represents T2DM mice treated with 80 mg/kg body weight surfactin after STZ injection. The levels of CAT (A), SOD (B), GSH-Px (C) and MDA (D). * indicate significant differences at $p < 0.05$, ** indicate significant differences at $p < 0.01$.

Figure S5. High performance liquid chromatography data. HPLC chromatogram of the standard of surfactin (A), HPLC chromatogram of surfactin sample 1 (B), HPLC chromatogram of surfactin sample 2 (C), HPLC chromatogram of surfactin sample 3 (D), standard curve of surfactin (E).

Figure S1. The western blot results for pancreas tissue. The control group represents normal mice fed a basic diet, the T2DM group represents mice with T2DM induced by a 60% fat diet and 30 mg/kg body weight STZ, and the surfactin group represents T2DM mice treated with 80 mg/kg body weight surfactin before STZ injection.

Figure S2. The western blot results for colon tissue. The control group represents normal mice fed a basic diet, the T2DM group represents mice with T2DM induced by a 60% fat diet and 30 mg/kg body weight STZ, and the surfactin group represents T2DM mice treated with 80 mg/kg body weight surfactin before STZ injection. The level of Caspase-1 (A), Cleaved-caspase-1(B), and Cleaved-caspase-1/Caspase-1. * indicate significant differences at $p < 0.05$, ** indicate significant differences at $p < 0.01$.

Figure S1

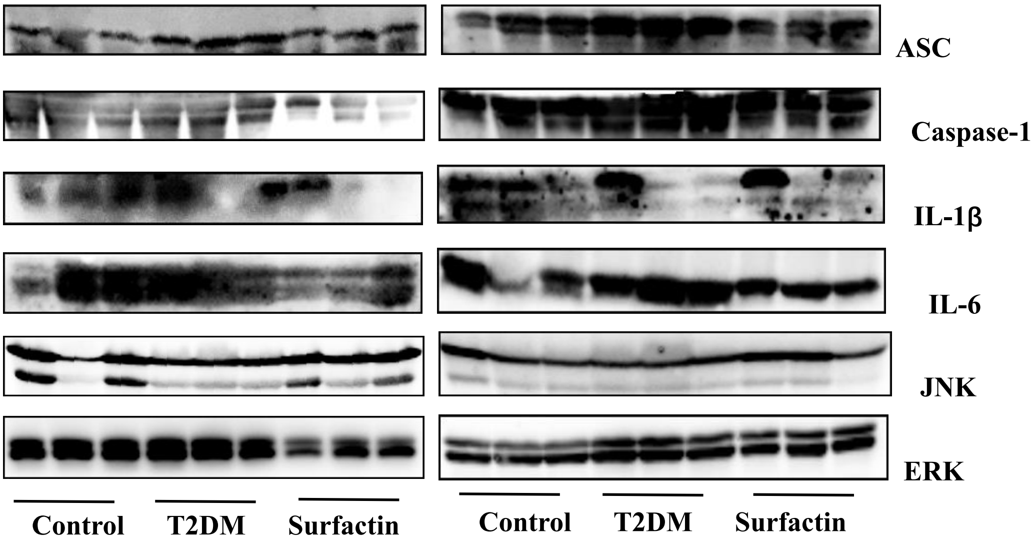

Figure S2

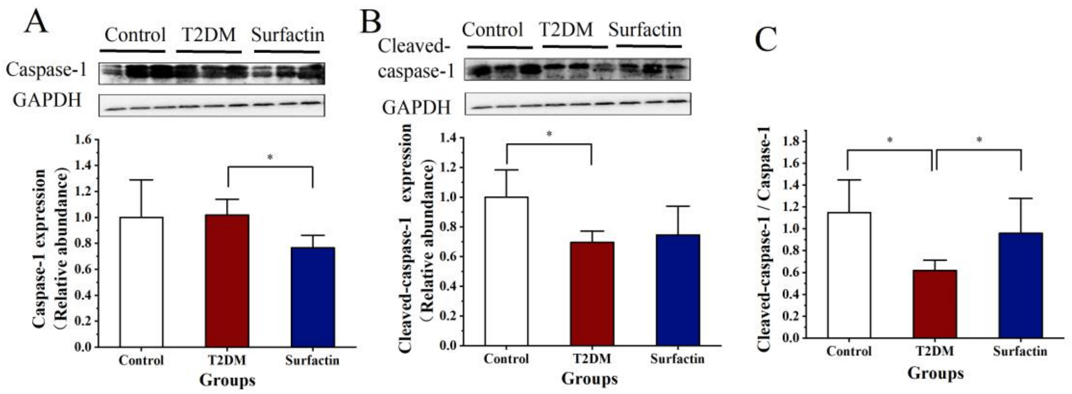

Figure S3

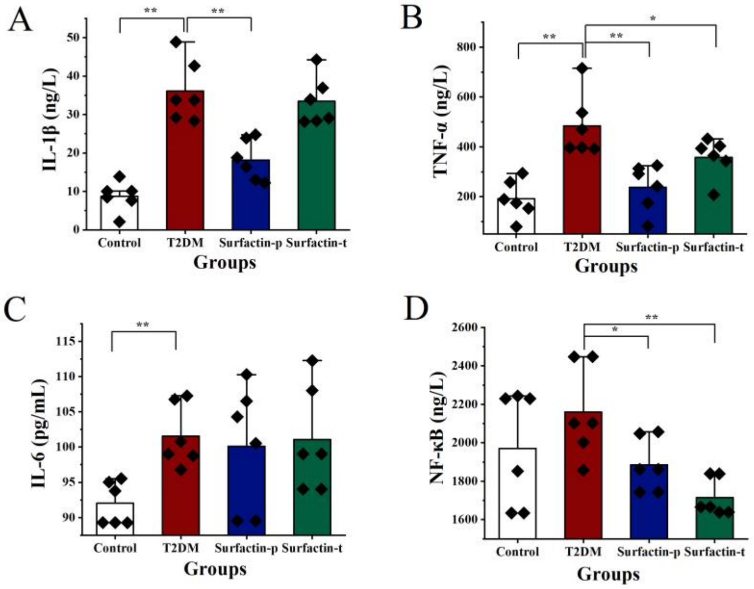

Figure S4

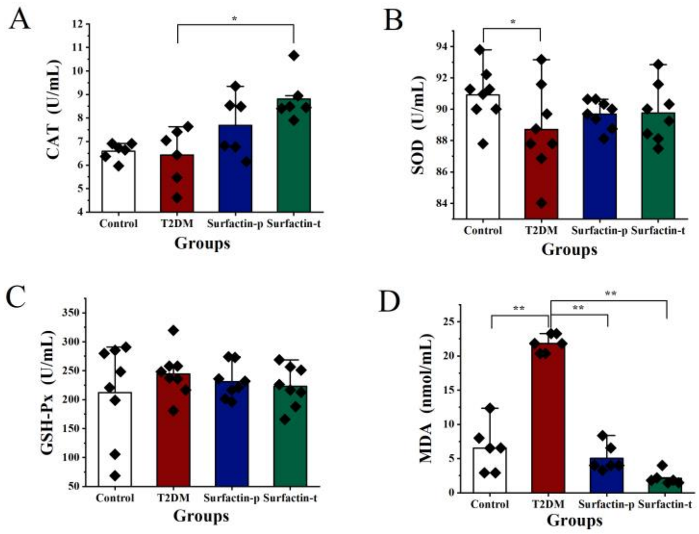

Figure S5

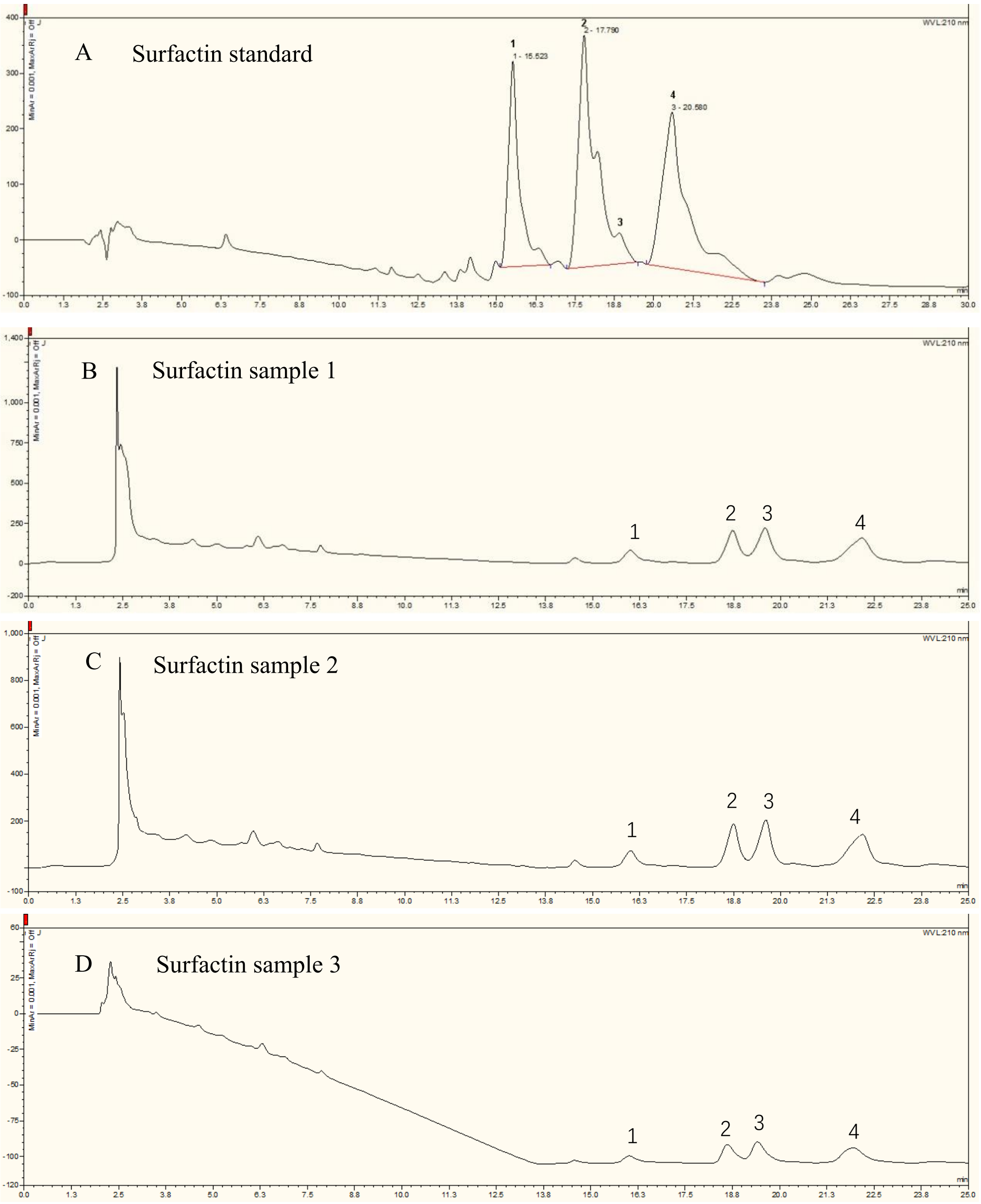
A
Surfactin standard
1 - 15.523
2 - 17.780
3 - 20.580
WVL:210 nm
B
Surfactin sample 1
C
Surfactin sample 2
D
Surfactin sample 3
min

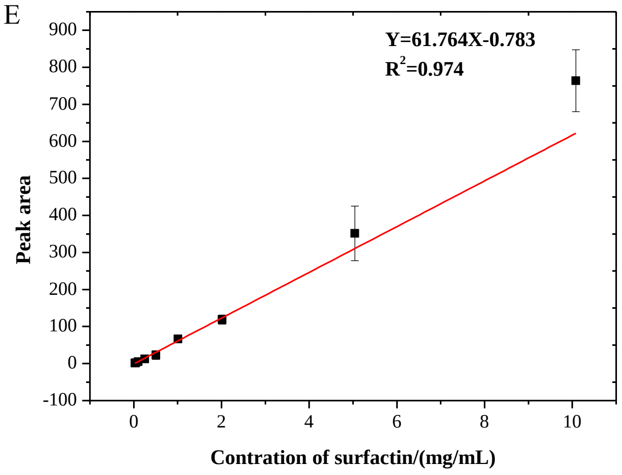

Standard curve of surfactin

Table S1. The raw data for pancreas protein.

**GAPDH**	Control	T2DM	Surfactin	Akt	Control	T2DM	Surfactin	p-Akt	Control	T2DM	Surfactin
**Relative**	190608	152770	140139		190977	36147	161376		117906	87116	128720
	174205	169887	199526		195973	315342	202685		76833	81161	141348
**grayscale**	171894	172182	215613		200941	37142	207869		54489	84228	84022
**Calculation formula= Target protein/GAPDH (a), the final result = a/control a, the result is as follows:**											
P-Akt/Akt	1.47812	5.77009	1.90969520	Akt	Control	T2DM	Surfactin	p-Akt	Control	T2DM	Surfactin
	0.93866	0.61620	1.66964842		0.91199	0.21536	1.048166		1.34803	1.24270	2.0016793
	0.64922	5.42935	0.96774349		1.02396	1.68955	0.924639		0.96115	1.04110	1.5438235
					1.06404	0.19634	0.877536		0.69080	1.06604	0.8492304
**GAPDH**	Control	T2DM	Surfactin	PI3K	Control	T2DM	Surfactin	p-PI3K	Control	T2DM	Surfactin
**Relative**	190608	152770	140139		12407	67825	9440		17665	10406	26335
	174205	169887	199526		53258	43093	38304		21405	20841	40544
	171894	172182	215613		45254	21475	36135		15911	41427	48548
**Calculation formula= Target protein/GAPDH (a), the final result = a/control a, the result is as follows:**											
p-PI3K/PI3K	2.93009	0.31573	5.74112192	PI3K	Control	T2DM	Surfactin	p-PI3K	Control	T2DM	Surfactin
	0.82711	0.99528	2.17830107		0.30796	2.10053	0.318705		0.90237	0.66322	1.8297297
	0.72356	3.96995	2.76489552		1.44644	1.20011	0.908284		1.19637	1.19445	1.9785167
					1.24558	0.59009	0.792921		0.90125	2.34265	2.1923457

Table S2 The raw data for colon protein.

**GAPDH**	Control	T2DM	Surfactin	Nf-κB	Control	T2DM	Surfactin	TNF-α	Control	T2DM	Surfactin
**Relative**	38995	46949	35867		25026	33960	13954		38943	64339	50118
	36052	39078	36307		22526	37995	16659		71250	83437	13094
**grayscale**	36171	41997	44725		20995	30067	10227		21533	18872	30670
**Calculation formula= Target protein/GAPDH (a), the final result = a/control a, the result is as follows:**											
				Nf-κB	Control	T2DM	Surfactin	TNF-α	Control	T2DM	Surfactin
					1.04238	1.17486	0.631903		0.83914	1.15150	1.1741303
					1.01484	1.57921	0.745256		1.66063	1.79408	0.3030397
					0.94276	1.16283	0.371402		0.50022	0.37758	0.5762103
GAPDH	Control	T2DM	Surfactin	IL-6	Control	T2DM	Surfactin	IL-1β	Control	T2DM	Surfactin
**Relative**	38995	46949	35867		17102	63660	30738		40474	34656	36260
**grayscale**	36052	39078	36307		32649	36077	13679		67287	21240	11608
	36171	41997	44725		8934	45249	10054		9355	48053	21621
**Calculation formula= Target protein/GAPDH (a), the final result = a/control a, the result is as follows:**											
				IL-6	Control	T2DM	Surfactin	IL-1β	Control	T2DM	Surfactin
					0.82688	2.55649	1.615791		0.98445	0.70013	0.9588748
					1.70743	1.74061	0.710343		1.77023	0.51552	0.3032468
					0.46568	2.03139	0.423831		0.24530	1.08525	0.4585161
**GAPDH**	Control	T2DM	Surfactin	IL-18	Control	T2DM	Surfactin	NLRP3	Control	T2DM	Surfactin
**Relative**	38995	46949	35867		165527	40629	89559		19382	19021	17285
	36052	39078	36307		146681	125438	32430		68529	20434	71704
	36171	41997	44725		39599	132918	81118		22547	46424	17824
**Calculation formula= Target protein/GAPDH (a), the final result = a/control a, the result is as follows:**											
				IL-18	Control	T2DM	Surfactin	NLRP3	Control	T2DM	Surfactin
					1.35355	0.27594	0.796212		0.49354	0.40229	0.4785346
					1.29735	1.02355	0.284820		1.88748	0.51923	1.9610653
					0.34909	1.00920	0.578338		0.61896	1.09764	0.3957253
GAPDH	Control	T2DM	Surfactin	TLR4	Control	T2DM	Surfactin	ASC	Control	T2DM	Surfactin
**Relative**	38995	46949	35867		46849	39187	43000		73532	62674	98405
	36052	39078	36307		67494	39006	47937		92845	103764	52413
	36171	41997	44725		28458	34352	37108		34838	111271	54938
**Calculation formula= Target protein/GAPDH (a), the final result = a/control a, the result is as follows:**											
				TLR4	Control	T2DM	Surfactin	ASC	Control	T2DM	Surfactin

		0.93366	0.64865	0.931694		1.04293	0.73833	1.5174455
		1.45490	0.77570	1.026078		1.42436	1.46860	0.7984351
		0.61142	0.63567	0.644788		0.53270	1.46539	0.6793811

Table S3 The raw data for serum.

GSP	Control	T2DM	Surfactin-p	Surfactin-t
	2539.08507	2455.47252	2850.73184	2751.91701
	2455.47252	2957.14781	2204.63487	2645.50104
	2212.23601	3147.17633	2508.6805	2698.70902
	2105.82004	3420.8174	2318.65198	2995.15351
	2584.69191	2751.91701	2493.47822	2364.25883
	2288.24742	2789.92271	2599.89419	2736.71473
**GHb**	680.5	715.5	470.5	665.5
	703	848	590.5	573
	633	875.5	423	665.5
	733	678	595.5	823
	685.5	773	773	800.5
	633	900.5	640.5	665.5
**Acrp30**	124.75224	69.22985	98.48358	86.84179
	101.17015	78.48358	100.27463	104.45373
	97.28955	85.94627	96.39403	88.93134
	79.97612	79.67761	91.3194	99.0806
	79.0806	92.21493	93.70746	112.81194
	81.17015	92.81194	97.58806	93.11045
**Insulin**	6.88694	5.63581	6.95519	8.02434
	7.02343	6.02252	7.27366	6.93244
	6.36374	6.59122	6.11351	6.75045
	7.22816	4.70314	6.36374	6.95519
	6.56847	6.25	6.68221	7.50114
	6.72771	6.72771	5.77229	5.34008
	6.97793	5.13535	7.81961	6.45473

	6.65946	8.00159	7.68312	7.79686
**HOMAIR**	1.79937	2.36852	1.99411	2.77514
	2.23056	2.19919	2.1143	2.54418
	1.75126	2.59648	2.10637	2.4005
	2.18139	2.36725	2.18185	2.28688
	2.08602	3.24154	1.60889	1.84126
	1.8617	2.5346	2.10637	2.15586
**GLP-1**	2.54802	2.9774	3.5452	3.33898
	2.88701	2.9548	3.7226	3.30508
	2.71751	3.63277	3.17684	3.50847
	3.19209	3.51977	4.35593	3.16949
	2.71751	3.35028	4.27684	3.50847
	3.19209	2.81921	4.15593	3.33898
**Blood glucose**	5.87863	8.22506	7.42609	7.78139
	7.66452	8.45881	6.16849	9.04834
	6.19187	8.08527	7.78139	8.00112
	6.79028	10.12099	8.50136	7.39804
	7.14558	8.43806	7.34661	9.94642
	7.75849	8.21697	6.27134	7.75802
	7.19233	8.42506	7.18766	7.51491
	6.29004	10.02099	6.16849	7.34194
